# A ZigBee-Based Location-Aware Fall Detection System for Improving Elderly Telecare

**DOI:** 10.3390/ijerph110404233

**Published:** 2014-04-16

**Authors:** Chih-Ning Huang, Chia-Tai Chan

**Affiliations:** Institute of Biomedical Engineering, National Yang-Ming University, No.155, Section 2, Linong Street, Taipei, 112 Taiwan; E-Mail: g39604001@ym.edu.tw

**Keywords:** fall detection, accelerometer, indoor positioning, ZigBee, pervasive healthcare

## Abstract

Falls are the primary cause of accidents among the elderly and frequently cause fatal and non-fatal injuries associated with a large amount of medical costs. Fall detection using wearable wireless sensor nodes has the potential of improving elderly telecare. This investigation proposes a ZigBee-based location-aware fall detection system for elderly telecare that provides an unobstructed communication between the elderly and caregivers when falls happen. The system is based on ZigBee-based sensor networks, and the sensor node consists of a motherboard with a tri-axial accelerometer and a ZigBee module. A wireless sensor node worn on the waist continuously detects fall events and starts an indoor positioning engine as soon as a fall happens. In the fall detection scheme, this study proposes a three-phase threshold-based fall detection algorithm to detect critical and normal falls. The fall alarm can be canceled by pressing and holding the emergency fall button only when a normal fall is detected. On the other hand, there are three phases in the indoor positioning engine: path loss survey phase, Received Signal Strength Indicator (RSSI) collection phase and location calculation phase. Finally, the location of the faller will be calculated by a *k*-nearest neighbor algorithm with weighted RSSI. The experimental results demonstrate that the fall detection algorithm achieves 95.63% sensitivity, 73.5% specificity, 88.62% accuracy and 88.6% precision. Furthermore, the average error distance for indoor positioning is 1.15 ± 0.54 m. The proposed system successfully delivers critical information to remote telecare providers who can then immediately help a fallen person.

## 1. Introduction

Because of increasing life expectancies and declining birth rates, many countries, particularly developed and developing countries, face aging population problems. Data from the International Database of the U.S. Census Bureau shows that in more developed countries the percentages of individuals over 65 years old were 6.9% to 28.7% of the population in 2013, and these percentages are expected to reach 17.0% to 59.0% in 2050. The aging trend is forecast to accelerate to a peak before 2035, and the world is expected to experience a significant acceleration in population aging in the future [1]. The number of elderly people who live alone or in the independent living communities has been increasing along with the aging population. Telecare automatically and remotely monitors real time emergencies and lifestyle changes over time in order to manage the risks associated with solitary living.

Some studies have found that approximately one-third of elderly people are involved in falls every year and about 10% of elderly fallers suffered multiple falls [2,3,4]. The World Health Organization (WHO) indicated that falls are the second leading cause of unintentional injury deaths for those over 65 years old worldwide. Falls and fall-induced injuries, which include fatal and non-fatal injuries, are important public health problems and threats for the elderly [5,6]. The annual medical costs associated with fall-related injuries among elderly people in the USA are approximately $0.2 billion for fatal injuries and $19 billion for non-fatal injuries [6]. Non-fatal injurious falls can not only cause disability or functional impairment, but also have psychological effects such as fear of falling again that reduce the range of Activities of Daily Living (ADLs) [4]. Effective fall management includes two strategies: fall prevention and fall detection. The fall prevention strategy corrects risk factors and situations or delivers targeted interventions to reduce the incidence of falls [7]. The most frequently mentioned physiological reasons for falls are loss of balance, medical conditions, muscle weakness and visual impairment, while environmental factors includes obstacles, the weather and lighting [8]. Unfortunately, aging factors cannot be fully eliminated, so an automatic fall detection system is essential for elder persons.

Recently, several well-known approaches for designing automatic fall detection systems have been developed that can be classified into three main categories according to the type of sensor used: video-based, acoustics-based and wearable sensor-based fall detection systems [9,10,11]. Anderson *et al*. constructed a three-dimensional representation of humans from silhouettes obtained from multiple cameras [9]. Zigel *et al*. used optical fiber sensors and acoustic sensors deployed beneath the floor to collect vibration signals generated by falls [10]. These two techniques perform well in controlled environments such as the laboratory, but these techniques are impractical for application outdoors or in real indoor environments like homes, hospitals, or care facilities. Image analysis can detect falls only when the fallers are in the line of vision of the camera, and the lighting and framing of image analysis must be modified and pre-processed before fall detection. The acoustic signal attenuation and the deployment density of acoustic sensors should be carefully considered. The wearable sensor-based fall detection approach is more attractive for elderly people because of privacy concerns and its flexibility.

Recent advances in Information and Communication Technologies (ICT), Wireless Sensor Networks (WSNs) and Micro-Electro-Mechanical Systems (MEMS) have enabled a variety of telecare applications. System integration for smart home telecare provides safety and security services based on automatic and remote activity monitoring [12]. Fall detection systems can use wearable sensors such as micro-mercury switches [13], optical sensors [13], gyroscopes or accelerometers [14] to collect body movement signals. Those signals are then processed in the wearable sensors, home server or cloud server by several fall-detection methods. Finally the fall alarm, location and faller information are transmitted through the WSNs, Internet or Global System for Mobile Communications (GSM) to the caregivers, doctors or families of the faller.

**Figure 1:**
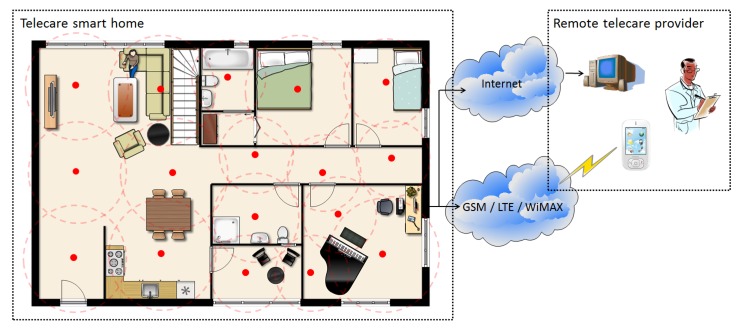
Scenario of ZigBee-based location-aware fall detection system.

This study uses a tri-axial accelerometer mounted on the waist to measure motion acceleration, and proposes a fall detection scheme and an indoor positioning engine to detect the fall and the faller position using the Received Signal Strength Indicator (RSSI). The proposed method can provide effective fall alarms and reliable indoor position information that not only reduce the fear of falling, but also can reassure the user about maintaining an independent lifestyle. Figure 1 shows the scenario of a ZigBee-based location-aware fall detection system for a telecare service that contains two subsystems, namely the fall detection and indoor positioning subsystems. This study combines the powerful features of each subsystem to let caregivers immediately identify accident locations. In the telecare smart home, the nodes are classified into four types: wearable sensor, RF generator, reference node, and gateway. In the event of a fall, the wearable sensor detects the event and transmits the fall alarm to the gateway to trigger the indoor positioning subsystem based on the reliable transmission protocol [15]. The server in the smart home calculates the location of the faller by the RSSI values broadcasted from the RF generator to all reference nodes and to the wearable sensor. The server at home can be connected using Wireless Wide Area Networks (WWAN) technology, such as Long Term Evolution (LTE) and Worldwide Interoperability for Microwave Access (WiMAX), GSM and Internet, to achieve a seamless platform for remote telecare monitoring. Finally, the fall alarm and faller information are transmitted to the remote telecare provider, and the caregivers can provide assistance.

The rest of this paper is organized as follows: Section 2 briefly introduces related fall detection and indoor positioning technologies. Section 3 then details the proposed ZigBee-based location-aware fall detection system. Using real data collected from ZigBee-based sensor networks, the experimental results demonstrate the capabilities of the proposed system. Section 4 gives a clear explanation and discussion. Finally, Section 5 addresses our conclusion.

## 2. Related Work

### 2.1. Fall Detection

Wearable sensor-based fall detection methods primarily comprise two types: threshold-based methods and machine learning methods [11,16]. The body experiences a fall in three stages: free fall, impact, and finally lying on the ground, which might generate lower peak acceleration values during the free fall, higher peak acceleration values at the moment of impact and a static acceleration value when lying on the ground. The simple threshold-based methods discriminate between the falls and ADLs when the peak values are below or above some threshold [17]. The advantages of simple threshold-based method are low computing complexity, and the algorithms can work on wearable sensors, but the ranges of peak acceleration values generated by falls and ADLs overlap, making it difficult to set an appropriate simple threshold. Furthermore, the threshold is also dependent on individual subjects and sensor location. Machine learning methods can overcome these disadvantages of threshold-based methods. Supervised learning algorithms train the classification using labelled fall and ADL data during the training period and thus identify individual falls during the classification period [18]. Unsupervised learning algorithms cluster the original data automatically using clustering algorithms instead of artificially labeled data before training [19]. Nevertheless, machine learning algorithms require an extended training period to ensure fall patterns are isolated from ADL patterns in the classification database. For a wearable sensor-based fall detection system, a reliable threshold-based method is more suitable since it has the advantages of fast response time and low sensor power consumption.

Wearing location is critical for wearable sensor-based fall detection algorithms. The most common wearing locations are waist, wrist, trunk, thigh and head. Some studies have aimed to evaluate the effectiveness of different wearing locations based on threshold-based fall detection algorithms. For example, Bourke *et al*. [17] used upper and lower fall thresholds to detect falls using tri-accelerometer sensors mounted on the trunk and thigh. The results showed that the 3.5 G upper fall threshold for the trunk had higher specificity than other situations and suggested the trunk was the optimum location for a fall sensor. Kangas *et al*. [20] compared different simple fall detection algorithms using accelerometers attached at the waist, wrist and head. The results ultimately indicated that the effective sensor location were the waist and head. The sensor at head level had the highest accuracy, but the usability and user’s acceptance should be considered in more detail. In conclusion, an accelerometer worn on the waist might be an optimal choice for a wearable sensor-based fall detection algorithm.

### 2.2. Indoor Positioning

For an indoor environment, several well-known positioning systems based on various technologies, such as InfraRed(IR), ultrasound, Radio-Frequency IDentification (RFID), Wireless Local Area Network (WLAN), Bluetooth, ZigBee, magnetic signals, camera *etc*., have been proposed. Furthermore, the position estimation algorithms are classified into four main categories: triangulation, fingerprinting, proximity and vision analysis [21,22]. Triangulation is the process of determining the target location using the geometric properties of triangles and measuring distances or angles to the target location from known reference points. The triangulation techniques utilize RSSI, Time of Arrival (ToA), Time Difference of Arrival (TDoA), Roundtrip Time of Flight (RToF) or Angle of Arrival (AoA) to estimate the target position. However, it is difficult to provide a standard radio propagation model in indoor environments owing to the severe multipath fading and environmental interference. The fingerprinting technique compares real-time RSSI values in the online position determination phase with the RSSI signal patterns saved during the offline training phase to obtain the estimation position. A target position is calculated by comparing current measurements with the pre-recorded radio map. The radio map comprises pre-recorded measurements of signal strength from reference locations, denoted as fingerprints. The proximity positioning scheme calculates the target position using the known reference node or an area such as a room, and it fulfills the requirement of room-based location service. The vision-based positioning system analyzes the image to detect the position of humans in the region monitored by the camera. The disadvantage of vision-based positioning system involves privacy problems; moreover, interference from lighting and the dynamically changing environment reduce the positioning accuracy. To simultaneously achieve high accuracy and low cost, RF technologies that include RFID, WLAN, Bluetooth and ZigBee have become increasingly popular for indoor location system.

The RF-based indoor location systems use the RSSI feature to estimate the distance between two objects, establish fingerprinting database or sense the proximity known reference tags. However, environmental interferences, such as multi-path fading, moving objects, temperature and humidity, can severely affect the accuracy of the triangulation technique; moreover the fingerprinting database is troublesome to implement. To avoid environmental influences and complexity of site survey work before positioning, Ni *et al*. [23] proposed a LANDMARC system that used real-time RSSI values of fixed reference tags and tracking tag received by the fixed RF readers to calculate the Euclidian distance between reference tags and tracking tag. The real-time RSSI of all devices suffered from the same noise influence, allowing the accommodation of environmental factors. The LANDMARC system selected the k-nearest reference tags to estimate the position of tracking tag. However, the variations in physical characteristics of tags and the dynamic indoor environment cause estimation bias.

## 3. ZigBee-based Location-aware Fall Detection System

**Figure 2:**
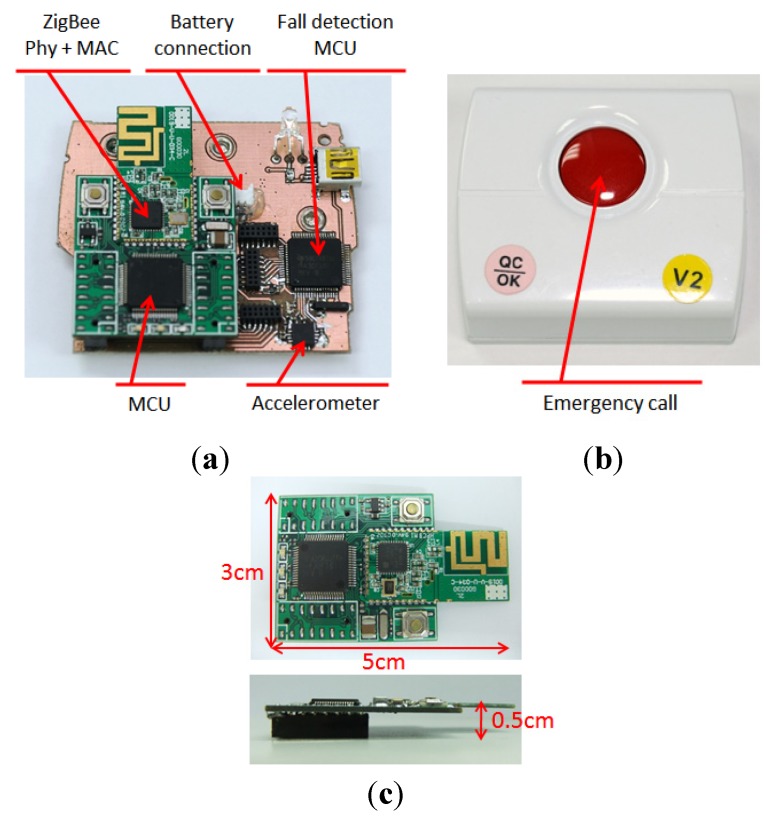
The hardware of wearable sensor and ZigBee module (**a**) The composition of wearable sensor. (**b**) The case of wearable sensor. (**c**) ZigBee module.

In the ZigBee-based location-aware fall detection system, the wearable sensor detects the fall event and transmits the fall alarm to the gateway to trigger the indoor positioning engine based on the reliable transmission protocol [15]. The nodes can be categorized into four types according to functions: wearable sensor, RF generator, reference node, and gateway, as shown in Table 1. All kinds of nodes use ZigBee module that comprises a MSP430 microcontroller and an UZ2400 ZigBee chip to transmit the signals. The UZ2400 transforms the power value (dBm) into the RSSI value linearly, the power value after transformation ranges from 0 to 255. The wearable sensor includes a ZigBee module and a motherboard with a MSP430 microcontroller, an ADXL 325 tri-axial accelerometer (±5 G). Except for the gateway connected with a computer directly, the power is supplied by the battery for the other three kinds of nodes. Figure 2 shows the ZigBee module and wearable sensor. The ZigBee module has dimensions 5 cm (length) × 3 cm (width) × 0.5 cm (height) and the wearable sensor in this case has dimensions 6 cm (length) × 5 cm (width) × 2 cm (height).

**Table 1:** The descriptions of nodes in the ZigBee-based location-aware fall detection system.

Nodes	Hardware	Functions
Wearable sensor	ZigBee module; Mother board; Battery	Detect fall; Receive signals from RF generators and send RSSI to computer through gateway
RF generator	ZigBee module; Battery	Send the broadcast signal to wearable sensor and reference nodes
Reference node	ZigBee module; Battery	Receive signals from RF generators and send RSSI to computer through gateway
Gateway	ZigBee module	Deliver the data from all the nodes to computer

**Figure 3:**
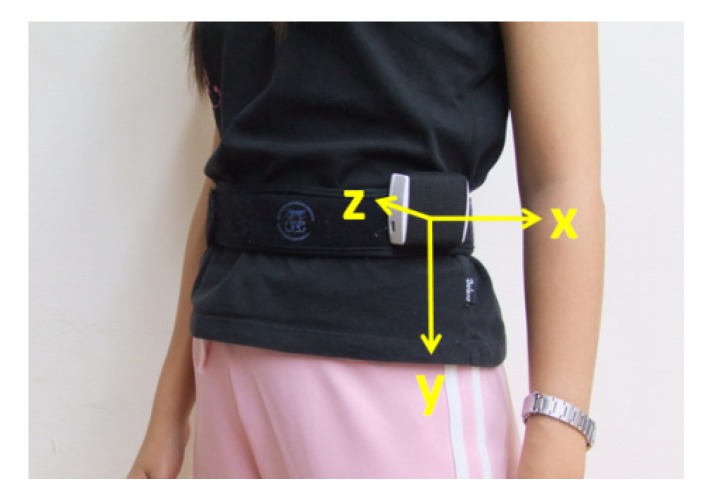
Wearing position and the axial direction of sensor.

The simulated falls were performed on a soft mat by nine healthy young subjects (62.89 ± 15.26 kg weight, 167.11 ± 8.1 cm height) wearing protectors. The volunteers wore the sensor on the left side of waist, and according to the placement of the tri-axial accelerometer, the *x*-axis represents the sagittal direction, the *y*-axis the vertical direction, and the *z*-axis the frontal side, illustrated in Figure 3.

The sample rate of the accelerometer is 200 Hz. The simulated falls occur in four main directions, front, posterior, left and right lateral falls, following seven kinds of ADLs. For example, the subject stands up and then falls forward. To clarify the effect between the wearing side (left) and the lateral falling side, two situations occur during lateral falls (left and right). There are 28 kinds of simulated fall types. On the other hand, this study also selects seven types of ADLs with normal or fast speed, such as standing up, sitting down, lying, walking, jumping, running, going up and down stairs. Table 2 lists the all characteristics of falls and ADLs.

**Table 2:** The characteristics of falls and daily activities.

**Daily Activities**	**Characteristics**			
Stand up	From sit		From squat	
Sit down	Normal		Fast	
Lie on the bed	Normal		Fast	
Walk	Normal		Fast	
Jump	On the ground		On the bed	
Go up and down stairs	Normal		Fast	
Run (18 m)	Normal speed			
**Activities before Falls**	**Fall Directions**			
Stand	Front	Posterior	Right lateral	Left lateral
Sit to stand	Front	Posterior	Right lateral	Left lateral
Stand to sit	Front	Posterior	Right lateral	Left lateral
Walk	Front	Posterior	Right lateral	Left lateral
Stoop	Front	Posterior	Right lateral	Left lateral
Jump	Front	Posterior	Right lateral	Left lateral
Walk backward	—	Posterior	Right lateral	Left lateral
Lie on the bed (25 cm height)	Turn the body then fall to the ground			

**Figure 4:**
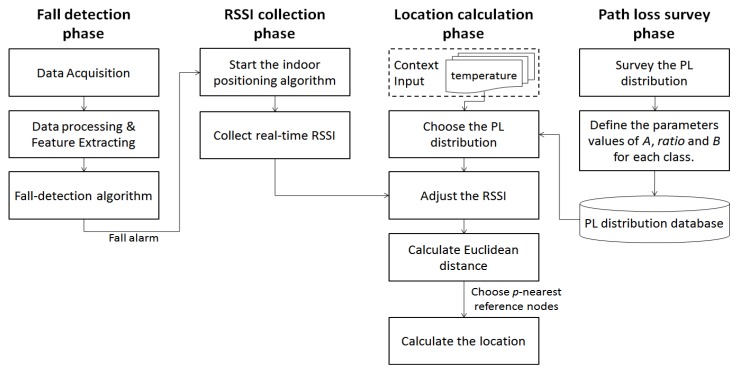
The flow diagram of ZigBee-based location-aware fall detection system.

The flow diagram of ZigBee-based location-aware fall detection system is shown in Figure 4. When falls can be detected in the fall detection phase, the fall alarm will start the indoor positioning engine that consists of three phases: path loss survey phase, RSSI collection phase and location calculation phase to detect the location of the faller in real time.

### 3.1. Fall Detection Phase

After receiving the raw data from the accelerometer, the data processing phase handles the analog to digital signal conversion and memory allocation. Furthermore, the results of the data processing and feature extraction phase are calculated using the fall-detection algorithm. The fall alarm can be classified into two kinds based on level of emergency. One is the normal fall alarm that occurs during non-serious falls, and in this case the user can cancel the alarm by pressing and holding the emergency fall button, like the red button in Figure 2b. The other is the critical fall alarm indicating the fall is sufficiently serious to cause fatal injury, and in this situation the faller requires immediate help.

**Figure 5:**
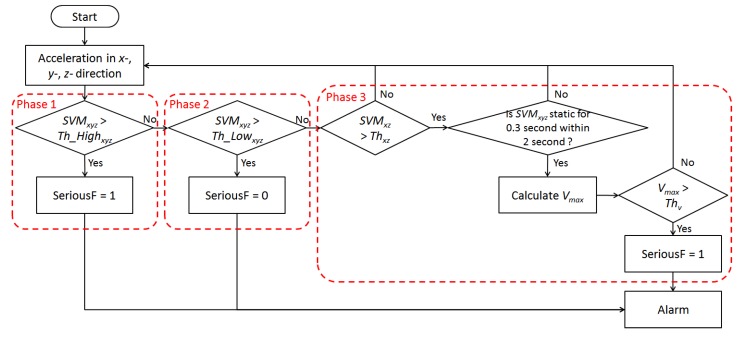
The fall detection algorithm.

The fall detection algorithm includes three phases, as shown in Figure 5. Phase 1 and Phase 2 use the upper peak acceleration to detect falls and Phase 3 evaluates the vibration by reference velocity. During the first phase, the Sum Vector Magnitude of tri-axial accelerations *(SVM_xyz_*) was adopted as the threshold in fall detection. Let *SVM_xyz_* be defined as:

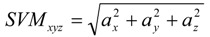
(1)
where *a_x_*, *a_y_* and *a_z_* are the accelerations of the *x*-axis, *y*-axis, and *z*-axis, respectively. If *SVM_xyz_* exceeds the maximum *SVM_xyz_* value of ADLs, the critical fall alarm is detected. The maximum *SVM_xyz_* value of ADLs is then set as the first fall detection threshold, *Th_High_xyz_*. In Phase 2, to avoid the users being helpless following minor falls, the maximum *SVM_xyz_* value of normal speed ADLs is set as the second fall detection threshold, *Th_low_xyz_*. However, some *SVM_xyz_* values of ADLs with fast speed exceed the *Th_low_xyz_*, so when normal fall alarms are detected, the user can cancel the alarm by pressing and holding the emergency fall button. Following the experimental results from the maximum *SVM_xyz_* value of ADLs with fast speed and the maximum *SVM_xyz_* value of normal speed ADLs, we set the *Th_High_xyz_* = 6 G and *Th_low_xyz_* = 3.5 G.

In the final phase, the variation of velocity on horizontal plane (*x*-*z* plane), *SVM_xz_*, that means the body tilt forward, backward or laterally is calculated by:

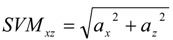
(2)

During the fall, the acceleration on horizontal plane will exceed 2 G. To distinguish falls from ordinary daily activities, the threshold *Th_xz_* is set to 2 G. Moreover, the reference velocity (*V_max_*) is defined as:

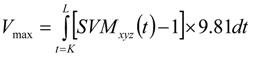
(3)
where the *K* is the time when *SVM_xz_* is larger than *Th_xz_*, and *L* is the occasion when the body is static for 0.3 s within 2 s after *SVM_xz _*is larger than *Th_xz_*. Before the integration, the acceleration component due to gravity (1 G) must be subtracted from *SVM_xyz_*. Regarding the accelerometer position, severe injuries might occur if the falling reference velocity is more than the threshold of *V_max_* (*Th_v_*). The threshold of the *V_max_* is set to 1.7 m/s that helps distinguish some violent daily activities from critical falls.

### 3.2. Path Loss Survey Phase

According to the characteristics of radio frequency signal, the power of the received signal decreases with distance. The log-distance Path Loss (PL) model describes the relationship between received power and distance, which is calculated as follows:

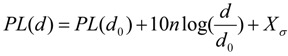
(4)
where the parameter *n* is called the PL exponent. Path loss can be represented by the path loss exponent, whose value depends on the specific propagation environment. And *n* = 2 is for propagation in free space. The *PL(d_0_)* is the received power from the transmitter at a known close distance *d_0_*, which is typically one meter for indoor system, and *X_σ_* denotes a zero mean Gaussian random variable that reflects the interference from indoor environment [24]. Unfortunately, the real received signal power fails to conform with the PL mode, and the PL distribution described by real signal presents a non-log-linear relationship between RSSI and distance. In the path loss survey phase the PL distribution of ZigBee module is surveyed in different contexts and the parameter values of each class are defined to increase positioning accuracy. According to the characteristics between RSSI and distance, the RSSI can be divided into four classes based on our previous research [25]:

- Class I: the RSSI value in Class I is measured within 0.5 m transmission distances.
- Class II: the RSSI value in this class is measured between 0.5 m to 2 m transmission distances. And the RSSI versus distance is near linear relationship in Class II.
- Class III: the RSSI in this class will be distributed at the distance from 2 m to 7 m.
- Class IV: when the distance far from 7 m and above, the RSSI values rapidly decay in Class IV.

The range of each class is defined for a temperature of 25 °C: Class I (144–255), Class II (112–143), Class III (60–111) and Class IV (0–60).

### 3.3. RSSI Collection Phase

In the RSSI collection phase, the home server gathers the RSSI from the RF generator to the wearable sensor and reference nodes, and these RSSI values are adjusted and calculated for the location of the wearable sensor during the location calculation phase. First, the wearable sensor triggers the RSSI collection phase by sending the fall alarm to the gateway. The gateway then asks RF generators in turn to send the broadcast message to all reference nodes and the wearable sensor. As soon as the wearable sensor and reference nodes receive the broadcast message, they save the value of RSSI into their memory. After all RF generators have sent the broadcast message, the wearable sensor and reference nodes will forward the RSSI values to the gateway. Finally, the location of the wearable sensor is calculated on the home server during the next phase.

### 3.4. Location Calculation Phase

After all the RSSI values are transmitted to the computer, the location of the wearable sensor is calculated during this phase. Suppose a situation involving *M* fixed reference nodes, *N* fixed RF generators and one wearable sensor installed in a telecare smart home. The RSSI vector of the wearable sensor is *W* = (*w_1_*, …, *w_N_*), and the corresponding RSSI vector of the *i*-th reference node is *R_i_* = (*r_i1_*, …, *r_iN_*). The Euclidean distance between the wearable sensor and the reference node *i* is then defined as:

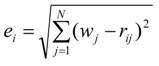
(5)

The wearable sensor has a Euclidean distance vector, *E* = (*e_1_*, …, *e_M_*), demostrating the similarity between the wearable sensor and all reference nodes. The smallest *e_i_* means the reference node *i* is the nearest reference node surrounding the wearable sensor. Based on our previous research [25], the RSSI should be adjusted before calculating the Euclidean distance, as follows:

*rss'* = *A* + *ratio*× (*rss* − *B*) (6)

The *rss* is the original RSSI value, and the *rss’* is named as the weighted RSSI value. The parameters *A*, *ratio* and *B* are decided according to different context conditions, such as the temperature and humidity. Table 3 lists the parameter values of *A*, *ratio* and *B* according to each class from the path loss survey phase at a temperature of 25 °C. The original RSSI in Equation (5) is replaced with the weighted RSSI, *W’* = (*w_1_’*, …, *w_N_’*) and *R_i_’* = (*r_i1’_*, …, *r_iN_’*), to obtain the adjusted Euclidean distance vector. Finally, the *p*-nearest reference nodes are selected to calculate the unknown position of the mobile node by:

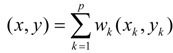
(7)
where *w_k_* denoted the weight of the *k*-th nearest reference node sorted by *E*, (*x_k_*, *y_k_*) is the coordinate of *k*-th reference node and (*x*, *y*) is the estimation position of the wearable sensor. The weight that depends on the *p*-nearest reference nodes is defined as:

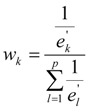
(8)
where *e^’^* is the modified Euclidean distance obtained using weighted RSSI vector of the wearable sensor and reference nodes. Equation (8) means the reference node with the smallest Euclidean distance has the largest weight.

**Table 3:** The parameter values at a temperature of 25 °C.

Class	A	ratio	B
Class I	176	0.05	144
Class II	112	0.3	112
Class III	60	0.6	60
Class IV	0	1	0

## 4. Results and Discussion

**Figure 6:**
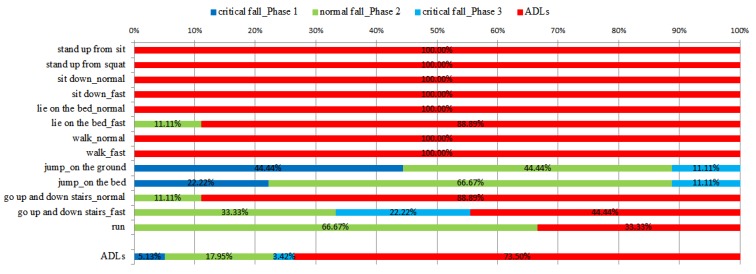
The detection results of ADLs using the proposed fall detection algorithm.

Figure 6 shows the experimental results of the ADLs using the proposed fall detection algorithm. The blue line and sky-blue line indicates the ADLs that are detected as critical falls, and the green line represents the ADLs that are detected as normal falls. Ideally, the ADLs should be classified as normal activities like the red line in Figure 6. This study finds that the ADLs acting in the house frequently, such as stand up from sitting and squatting, sit down, lie on the bed, walk and go up and down stairs with normal speed, have just two false alarm incidents in our fall detection algorithm. The false alarms happened on lying on the bed with fast speed and going up and down stairs with normal speed which are recognized as normal falls. If an ADL is recognized as a normal fall (Phase 2), users can cancel the alarm themselves to reduce the burden on remote telecare providers. Some acute ADLs will be detected as critical falls, for example, jumping, and going up and down stairs fast, but an elderly person seldom performs such acute ADLs at home. For ADLs acting in the house frequently, there exist only a 2.46% probability of normal falls, and 97.54% probability of ADLs. For all ADLs, there exist an 8.55% probability of false alarms, 17.95% probability of normal falls, and 73.5% probability of ADLs.

**Figure 7:**
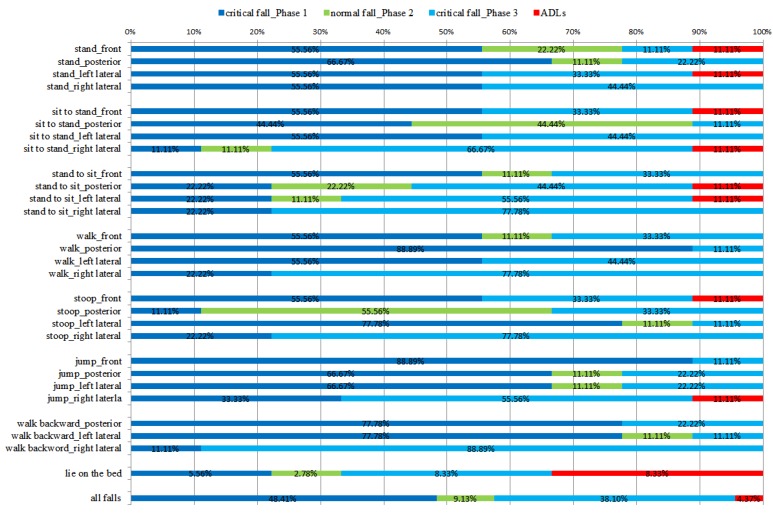
The detection results of falls using the proposed fall detection algorithm.

On the other hand, for fall detection results, Figure 7 presents the results of falls calculated using the proposed fall detection algorithm. Most falls can be detected as critical falls (the blue and sky-blue line), and about half of the critical falls are recognized during Phase 1, which means there exists a 48.41% probability that the acceleration of falls exceeds the maximum acceleration value of ADLs. Especially when the falls follow walking or jumping, there is about 60% probability to detect falls in Phase 1 that means the activities with speed before falling would increase the maximum acceleration value as the moment of impact. The falls that involve indirectly falling from a standing posture are frequently recognized during Phase 3, for example moving from standing to sitting followed by a fall has a 52.78% probability of being detected in Phase 3. If the system uses only the single threshold fall detection algorithm proposed by Bourke *et al*. [17] to detect falls, the falls detected in Phase 3 will be judged as ADLs that will have numerous false alarms. The high rate of false alarms reduces user’s acceptance of the system. The influence of wearing position on lateral falling direction is notable. Based on the same fall posture, more right lateral falls are identified in Phase 3 than left lateral falls. Because the sensor is worn on the left waist, the sensor impacts the ground directly during left lateral falls, which induces higher acceleration. Not only the activities before falling but also the sensor wearing position would affect the acceleration value during falling. In conclusion, the fall detection algorithm had an accuracy of 88.62%, precision of 88.6%, sensitivity of 95.63%, and specificity of 73.5% for all ADLs and falls. The results from ADLs acting in the house frequently and falls showed accuracy of 96.10%, precision of 99.18%, sensitivity of 95.63%, and specificity of 97.53%. The proposed three-phase fall detection algorithm can effectively detect the falls.

The indoor positioning experiment is performed in a 11 m (length) × 5.75 m (width) classroom. The gateway is located in the middle and the 18 reference nodes are fixed in the classroom at intervals of 2 m. Furthermore, five RF generators are set symmetrically between reference nodes. The error distance, *err*, can illustrate the performance based on the coordinate of the mobile node (*x_0_*, *y_0_*), and the estimation result (*x*, *y*) is defined as:

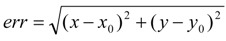
(9)

One of the key issues affecting the estimation position is to determine the optimal number *p* of nearest reference node(s) for Equations (7) and (8). Based on a previous study [23,25], setting *p* = 4 minimizes the error distance. The average error distance is 1.15 ± 0.54 m, the maximum error distance is 1.64 m and the minimum error distance is 0.24 m. Comparing the results from the original signals without weighted RSSI, the indoor positioning scheme we proposed reduces 0.47 m error distance averagely. In short, the indoor positioning algorithm can significantly improve the accuracy of indoor location.

## 5. Conclusions

Living in a telecare smart home has become popular among geriatric retirees. Unintentional falls pose a major health threat to the elderly and can result in severe injuries. An effective location-aware fall detection system for elderly telecare is important for delivering adequate and immediate medical support and to dramatically reduce medical care costs. Moreover, such a system can reduce the fear of falling and reassure the user that they can maintain their independence. This study uses a waist-mounted sensor node with a tri-axial accelerometer to monitor the movement data of the human body, and proposes a fall detection algorithm for analyzing motion patterns that can distinguish falls from ADLs. Furthermore, in the event of a fall, the location-aware mechanism starts the indoor positioning engine, and then provides immediate indoor position information to caregivers. The experimental results have demonstrated that the proposed algorithm has high accuracy for fall detection, and this study also designed a normal fall alarm to avoid a minor falls from rendering the victim helpless. The accuracy of the location information satisfies the requirements of location awareness. The proposed system delivers critical information to remote telecare providers and improves the quality of elderly care.
